# Use of prehospital, hospitalization and presence of sequelae and/or disability in road traffic injury victims in Brazil

**DOI:** 10.1371/journal.pone.0249895

**Published:** 2021-04-16

**Authors:** Gabriela Camargo Tobias, Polyana Maria Pimenta Mandacarú, Rafael Alves Guimarães, Otaliba Libânio Morais Neto

**Affiliations:** 1 Institute of Tropical Pathology and Public Health, Federal University of Goiás, Goiânia, Goiás, Brazil; 2 Faculty of Nursing, Federal University of Goiás, Goiânia, Goiás, Brazil; South China University of Technology, CHINA

## Abstract

**Objective:**

To estimate the prevalence and analyze the association between sociodemographic and behavioral variables with the use of prehospital care, hospitalization and sequelae and/or disability in victims of road traffic accidents victims in Brazil.

**Methods:**

Data from the National Health Survey conducted in 2013 in Brazil were used. Data were collected through a direct household survey. The research sample consisted of 1,840 individuals who reported road traffic accidents in the previous 12 months. Poisson regression analysis was used to evaluate the factors associated with the use of prehospital care services, hospitalization, and the presence of sequelae and/or disability.

**Results:**

The prevalence of road traffic accidents victims who received prehospital care was 13.0% (95% Confidence Interval [95% CI]: 10.3–16.3) and the factors associated with this outcome were: residing in the Northeast or North region of Brazil; residing in rural areas; and being a motorcycle occupant at the moment of the road traffic accident. The frequency of hospitalization was 7.7% (95% CI: 6.0–10.0) and the associated factors were: age between 40 and 59 years; being a motorcycle occupant or pedestrian and having received on-site care at the moment of the road traffic accident. The prevalence of sequelae and/or disability was 15.1% (95% CI: 12.5–18.2) and the associated factors were: age range between 30 and 39 years or 40 and 59 years; being a motorcycle occupant, being a pedestrian or belonging to other category of modes of transport and having received on-site care at the moment of the road traffic accident.

**Conclusion:**

The study allowed to evaluate the factors associated with prehospital care, hospitalization and presence of sequelae and/or disability in the victims of road traffic accident and the results can guide the implementation of interventions that prioritize the population exposed to the highest risk of road traffic accident injuries and with less access to prehospital and hospital care services in Brazil.

## Introduction

Injuries caused by Road Traffic Accidents (RTA) are a serious and complex public health problem, especially in low- and middle-income countries. Increased motorization rates associated with poor road infrastructure and the expansion of unsafe modes of transport (e.g., motorcycles), as well as risk behaviors (e.g., drinking and driving and excessive speed), are the main determinants of increased deaths and sequelae and/or disabilities caused by RTA [1]. It is estimated that 1.2 million deaths occur due to RTA injuries worldwide and that 50 million people are injured due to these problem [2].

In Brazil, approximately 40,000 people died each year in RTA and more than 200,000 people evolved with serious injuries in 2017 [3]. According to data from the Institute of Health Metrics and Evaluation (IHME), among South American countries, Brazil presents the second highest rate of Disability-Adjusted Life Years (DALY) per RTA (1,230/100 thousand inhabitants), behind only Paraguay (1,270/100 thousand inhabitants) [4]. In terms of costs, accidents in highways and urban roads in Brazil represent a total cost for society of approximately R$ 40.0 billion and R$ 10.0 billion per year, respectively. Most of this cost is related to loss of productivity and hospital costs, which depending on the severity of the injuries may be higher, especially in fatal cases [5].

The focus for addressing this serious public health problem and reducing the deaths caused by RTA are actions to promote traffic safety aimed at building safe roads and means of transportation and promoting safe behaviors by road users [6]. Although safety interventions are critical to reducing road traffic injuries and deaths, access to prehospital care (PHC) can have an impact on reducing RTA disabilities, sequelae, and lethality [7]. Studies have shown that early quality PHC (within the first hour of injury) and hospital care prevent and reduce the probability of death and sequelae that are potentially preventable due to RTA [8, 9].

In the context of a decentralized health system, such as Brazil’s, regular national assessments of the health care received by RTA victims who had injuries guide the adoption of best practices. They identify the bottlenecks in the health promotion, prevention and care related to RTA. Most studies about RTA focus on mortality or hospital admissions. Few studies have analyzed the prevalence and/or determinants of injuries that do not lead to hospitalization. The 2013 National Health Survey (NHS) filled this knowledge gap and provided a more complete picture of the magnitude, characteristics and modes of transport used by victims, as well as the analysis of factors associated with the occurrence of injuries, sequelae and/or disabilities, and the use of health care services.

The objective of this study was to estimate the prevalence and analyze the association between sociodemographic and behavioral variables with the use of PHC, hospitalization and sequelae and/or disability in victims of road traffic accidents victims in Brazil.

## Material and methods

### Design and data

Data from the NHS, a national population survey conducted by the Brazilian Institute of Geography and Statistics and the Ministry of Health in 2013, were used in the present study. The population was composed of residents of private households from the 27 federation units of Brazil. The sample consisted of 60,198 individuals aged 18 or over, randomly selected, among adults living in the household. The research presents statistical power for inferences in Brazil, Federative Units, and capital cities [10–12].

### Sampling and data collection

The sampling strategy of the NHS consisted of a complex sample in three stages: (i) primary sampling unit, corresponding to census tracts of the municipalities; (ii) secondary sampling unit, corresponding to households, and (iii) tertiary sampling unit, corresponding to individuals aged 18 years or older. In each stage, the sample units were randomly selected. The probability of selecting each individual aged 18 years and older within a household was weighted by household, adjusted by nonresponse rate, sex, and age calibration by the total population. Data was collected from the selected participants by home interviews. Details of the sampling design and sample size are available in previous studies [10–12].

### Variables

How many crashes had they experienced in that period, what was the mode of transport they used, and whether they were a pedestrian, driver, or passenger at the time of the most serious episode.

The NHS questionnaire asked participants whether they had been involved in a RTA that had resulted in a non-fatal injury in the last 12 months; if so, the analyzed variables were: (i) gender (male or female); (ii) age, categorized as 18–29 years, 30–39 years, and 40–59 years and 60 years and over; (iii) self-declared skin color/race (white; brown; black; or other, encompassing Brazilian Native and Asian); (iv) schooling (higher education or post-graduation, high school, elementary school, and less than elementary school); (v) married/partner (yes or no); (vi) current drinker, defined as alcohol use one or more times in the last month (yes or no); (vii) Binge drinking, defined as consumption of five or more units of alcohol for men or four or more for women on a single occasion in the last 30 days (yes or no); (viii) Drinking and driving (yes or no) in the last 12 months; (ix) Time interval between the accident and the first care measure (<60 or ≥60 minutes); (x) Macro-region of residence (South, Southeast, Central West, North or Northeast); (xi) Area of residence (capital, municipality of the metropolitan region of the capital, or other municipalities of the Federation Unit); (xii) Type of area of residence (urban or rural); (xiii) Condition of victim at the time of the accident (driver or non-driver); (xiv) Modes of transport (motorcycle occupant, car occupant, pedestrian, or other modes of transport, including bus drivers or passengers, truck drivers or passenger, cyclists and occupants of other modes of transport); (xv) Whether the victim stopped performing usual activities (yes or no); (xvi) Whether the victim received care on the site of the RTA (yes or no); (xvii) place where the victim received the first health care measures (on-site, primary health care unit, outpatient emergency unit, public hospital, private hospital, private emergency unit or others); (xviii) Who provided care at the scene of the accident (Emergency Mobile Assistance Service—SAMU), ambulance/fire and rescue service, rescue service of the private sector, or rescue service of private highway concessionaire companies).

The present study evaluated three outcomes: (i) PHC: individuals who presented injuries caused by RTA and who received emergency mobile prehospital care at the site of the accident (for this outcome, the prevalence of RTA victims was estimated according to the following variables: sex, age, race/skin color, schooling, married/partner, macro-region, area of residence, type of area, and modes of transport); (ii) hospitalization: people hospitalized for 24 hours and more (for this outcome, the prevalence of RTA victims was estimated according to the following variables: sex, age, race/skin color, schooling, married/partner, macro-region, current drinker, binge drinking, drinking and driving, area of residence, type of area, modes of transport, and PHC); (iii) presence of sequelae and/or disability resulting from RTA (for this outcome, the prevalence of RTA victims was estimated according to the following variables: sex, age, race/skin color, schooling, married/partner, macro-region, current drinker, binge drinking, drinking and driving, area of residence, type of area, modes of transport, and PHC).

### Statistical analysis

In the descriptive analysis, the sociodemographic and behavioral characteristics and the care received by the RTA victims were evaluated. The percentages and respective 95% confidence intervals (95% CI) were estimated.

For the analysis of factors associated to the three outcomes (PHC, hospitalization, and presence of sequelae and/or disabilities), bivariate and multivariate Poisson regression models were used [13–16]. Bivariate Poisson analysis was conducted to establish the bivariate relationship between each independent variable investigated and the outcome variables. The results of this analysis were presented as Crude Prevalence Ratio (PR) and respective 95% CI. Next, variables with a p-value in the bivariate analysis were included in multiple Poisson regression models to adjust for confounding variables. The results of the multivariate analysis were presented as Adjusted Prevalence Ratio (aPR) and 95% CI. The presence of multicollinearity was tested by analyzing the correlation matrix between the independent variables. Pearson’s correlation coefficient varies from -0.120 to 0.210, suggesting the absence of multicollinearity between variables (r<0.6). Statistical significance was established by Wald’s statistic. Associated factors were those that presented a critical value of p<0.05.

### Ethical aspects

The study was approved by the National Ethics Research Committee, protocol number 328.159, of June 26, 2013. Written consent was obtained from all participants.

## Results

Of the total number of adults who participated in the research (n = 60,198), 1,840 (3.1%; 95% CI: 2.8–3.3) reported a history of RTA injuries in the last 12 months.

Table 1 shows the sociodemographic and behavioral characteristics of the victims of injuries resulting from RTA. The predominant age group of the victims was 18–29 years (43.4%). The most of the victims were male (69.0%) lived with a marriage mate/partner (54.3%). Of the total number of participants with a history of RTA injuries, 41.2% and 46.8% declared to be white and brown, respectively. About schooling, 50.3% reported high school or higher education. Regarding the place of residence of the victim, the highest percentages of RTA were observed in urban areas (85.2%), in the Southeast (34.4%) and Northeast (29.5%) regions, and in municipalities out of the metropolitan regions of the country (66.2%).

**Table 1:** Sociodemographic and behavioral characteristics of RTA victims. National Health Survey, Brazil, 2013.

Variables	n = 1,840	%	95% CI^1^
**Sex**			
Female	572	31.0	27.7–34.7
Male	1,268	69.0	65.3–72.3
**Age (years)**			
18–29	798	43.4	39.3–47.5
30–39	527	28.6	25.3–32.3
40–59	409	22.2	19.2–25.6
≥ 60	106	5.8	4.3–7.7
**Race/skin color (self-reported)**			
White	758	41.2	36.8–41.7
Brown	198	46.8	42.6–51.1
Black	862	10.8	8.5–13.5
Others (Asian and Brazilian Native)	22	1.2	0.7–1.9
**Schooling**			
Higher education or post-graduation	212	11.5	9.0–14.6
High school	715	38.8	35.5–42.3
Elementary School	345	18.8	15.9–21.9
Less than elementary school	568	30.9	27.1–34.9
**Married/partner**			
Yes	999	54.3	50.2–58.3
No	841	45.7	41.7–49.8
**Area of residence**			
Capital	408	22.2	19.8–24.8
Metropolitan region	213	11.6	9.7–13.7
Other municipalities of the Federation Unit	1,219	66.2	62.9–69.4
**Type of area**			
Urban	1,568	85.2	82.4–87.6
Rural	272	14.8	12.4–17.6
**Macro-region**			
Southeast	633	34.4	30.2–38.9
South	255	13.8	11.2–16.9
Midwest	193	10.5	8.9–12.3
Northeast	542	29.5	26.3–32.8
North	217	11.8	10.0–13.8
**Current drinker**^**2**^			
Not	1,053	57.2	53.2–61.2
Yes	787	42.8	38.8–46.8
**Binge drinking**^**2**^			
No	1,340	72.9	69.1–76.3
Yes	500	27.1	23.7–30.9
**Drinking and driving**^**3**^			
No	1,581	85.9	82.9–88.5
Yes	259	14.1	11.5–17.1
**Condition of victim at the time of the accident**			
Driver	1,280	69.6	66.0–73.0
Non-driver	560	30.4	27.0–34.0
**Modes of transport**			
Car occupant	496	26.9	23.5–30.6
Motorcycle occupant	1,069	58.1	54.1–62.0
Pedestrian	89	4.9	3.7–6.3
Others	186	10.1	7.9–12.9
**Stopped performing usual activities due to the accident**			
No	972	52.8	48.8–56.8
Yes	868	47.2	43.2–51.2

^1^95% Confidence Interval

^2^In the last month

^3^In the last 12 months.

Regarding the behavioral characteristics of the victims, it was observed that 57.2% of the victims had consumed alcohol in the last 30 days; the prevalence of binge drinking was 27.1%; 14.1% reported drinking and driving in the last 12 months.

The motorcycle was the main mode of transport used by most of the RTA victims (58.1%), and in more than one third of the accidents (69.6%), the victim was the driver of the vehicle.

With regard to health care, 52.4% of the victims received some type of care due to the RTA. Theses, the assistance occurred mainly in public emergency care unit (29.2%), The SAMU was the main responsible for the care of the 239 victims who received care at the site of the accident (57.1%). The time between the accident and the first care measure was equal to or greater than 60 minutes for most victims (79.2%) (Table 2).

**Table 2:** Care characteristics of RTA victims. National Health Survey, Brazil, 2013.

Variables	N = 1,840	%	95% CI^1^
**Received some type of care due to the accident**			
No	876	47.6	43.3–52.0
Yes	964	52.4	48.0–56.7
**Site of first care received**^**2**^ **(n = 964)**			
Site of the accident	239	24.9	20.0–30.4
Basic Health Unit	128	13.3	9.8–17.7
Public Emergency Care Unit	281	29.2	24.8–34.0
Public hospital	215	22.4	17.8–27.6
Private clinic	48	4.9	2.7–8.9
Private Emergency Care Unit	49	5.0	3.1–8.2
Others	4	0.3	0.1–0.8
**Time interval between the accident and first care measure (minutes)**^**2**^ **(n = 964)**			
< 60	201	20.8	16.5–25.8
≥ 60	763	79.2	74.1–83.4
**Who provided care at the site of the accident**^**3**^ **(n = 239)**			
SAMU	137	57.1	44.2–69.0
Ambulance/ fire and rescue service	73	30.6	19.4–44.7
Private Rescue Service	15	6.2	3.2–11.6
Rescue service of private highway concessionaire companies	14	6.1	2.2–15.6

^1^95% Confidence Interval

^2^Regarding the total number of individuals who received some type of care due to the accident

^3^Regarding the total number of individuals who received care at the site of the accident.

The prevalence of victims who received PHC, who were hospitalized for 24 hours or more, and who had sequelae/disability was 13.0%, 7.7% and 15.1%, respectively (Table 3).

**Table 3:** Prevalence of prehospital care, hospitalization, and sequelae and/or disability in RTA victims. National Health Survey, Brazil, 2013.

Variables	n = 1,840	%	95%CI^1^
**Received prehospital care**			
No	1,601	87.0	83.7–89.7
Yes	239	13.0	10.3–16.3
**Hospitalization**			
No	1,698	92.3	90.0–94.1
Yes	142	7.7	5.9–10.0
**Sequelae and/or disability**			
No	1,562	84.9	81.8–87.5
Yes	278	15.1	12.5–18.2

^1^95% Confidence Interval.

The factors associated with PHC at the site of the RTA in the multiple regression analysis were: residing in the Northeast (aPR = 0.48, 95% CI: 0.27–0.84) or North (aPR = 0.33; 95% CI: 0.17–062); living in the rural area (aPR = 0.28, 95% CI: 0.14–0.53) and being a motorcycle occupant (aPR = 1.86, 95% CI: 1.07–3.27) (Table 4).

**Table 4:** Prevalence and factors associated with prehospital care in victims of RTA injuries. National Health Survey, Brazil, 2013.

Variables	Prevalence of PHC (n = 239)			Bivariate analysis			Multivariable analysis^3^		
	n/Total	%	(95% CI)^1^	Prevalence Ratio	(95% CI)^1^	p-value^2^	Adjusted Prevalence Ratio	(95% CI)^1^	p-value^2^
**Sex**									
Female	81/572	14.2	(10.0–19.4)	1.00					
Male	158/1,268	12.5	(9.2–16.6)	0.87	(0.55–1.37)	0.565	0.79	(0.52–1.20)	0.274
**Age (years)**									
18–29	98/798	12.3	(8.1–18.4)	1.00					
30–39	71/527	13.5	(9.4–19.1)	1.09	(0.63–1.89)	0.740	1.27	(0.76–2.10)	0.352
40–59	61/409	14.9	(9.8–22.1)	1.21	(0.67–2.16)	0.518	1.42	(0.87–2.30)	0.153
≥ 60	9/106	8.5	(3.5–19.2)	0.69	(0.26–1.79)	0.445	0.89	(0.36–2.18)	0.807
**Race/skin color (self-report)**									
White	84/758	11.1	(7.6–16.0)	1.00					
Brown	22/198	15.1	(10.7–20.9)	1.35	(0.84–2.17)	0.201	1.70	(0.96–3.03)	0.068
Black	130/862	11.2	(5.4–21.8)	1.00	(0.45–2.25)	0.982	1.16	(0.50–2.70)	0.723
Others (Asian and Brazilian Native)	3/22	11.2	(3.7–30.7)	1.03	(0.33–3.18)	0.954	1.59	(0.52–4.91)	0.411
**Schooling**									
Higher education or post-graduation	28/212	13.5	(6.3–26.7)	1.00			1.00		
High school	93/715	13.5	(8.6–19.1)	0.96	(0.39–2.36)	0.929	1.12	(0.52–2.41)	0.770
Elementary School	48/345	13.9	(7.8–23.5)	1.02	(0.40–2.58)	0.959	1.03	(0.46–2.28)	0.937
Less than elementary school	70/568	12.3	(8.7–17.1)	0.91	(0.40–2.05)	0.823	1.06	(0.49–2.30)	0.868
**Married/partner**									
Yes	128/999	12.8	(9.7–16.8)	1.00					
No	111/841	13.2	(9.0–19.0)	1.03	(0.64–1.64)	0.894			
**Macro-region**									
Southeast	100/633	15.9	(10.3–23.6)	1.00					
South	45/255	17.6	(9.5–30.3)	1.10	(0.54–2.26)	0.782	1.29	(0.65–2.54)	0.458
Midwest	32/193	16.9	(11.8–23.6)	1.06	(0.62–1.82)	0.820	0.80	(0.44–1.45)	0.473
Northeast	48/542	8.8	(6.1–12.4)	0.55	(0.32–0.95)	0.033	0.48	(0.27–0.84)	0.010
North	14/217	6.3	(3.7–10.6)	0.39	(0.20–0.77)	0.007	0.33	(0.17–0.62)	0.001
**Area of residence**									
Capital	59/408	14.5	(11.0–19.0)	1.00					
Metropolitan region	25/213	11.8	(7.4–18.2)	0.80	(0.47–1.37)	0.434			
Others	155/1,219	12.7	(9.1–17.5)	0.87	(0.57–1.34)	0.542			
**Type of area**									
Urban	228/1,568	14.5	(11.4–18.3)	1.00					
Rural	11/272	4.2	(2.6–6.7)	0.28	(0.16–0.51)	<0.001	0.28	(0.14–0.53)	<0.001
**Modes of transport**									
Car occupant	51/496	10.4	(6.1–17.1)	1.00					
Motorcycle occupant	152/1,069	14.2	(10.6–19.0)	1.37	(0.75–2.49)	0.296	1.86	(1.07–3.22)	0.026
Pedestrian	18/89	20.4	(11.4–33.8)	1.97	(0.92–4.19)	0.078	2.18	(0.91–5.26)	0.080
Others	18/186	9.4	(4.8–17.7)	0.90	(0.39–2.09)	0.820	0.92	(0.40–2.10)	0.856

^1^ 95% Confidence Interval

^2^Chi-square Wald’s test

^3^Model adjusted by sex, age, race/skin color, schooling, married/partner, macro-region, type of area and modes of transport.

The factors associated with hospitalization in the multiple regression model were: age group between 40 and 59 years (aPR: 2.46; 95% CI: 1.16–5.23); being a motorcycle occupant (aPR: 2.91, 95% CI: 1.43–5.93) or pedestrian (aPR: 3.63, 95% CI: 1.78–7.38) and having received PHC (aPR: 3.29, 95% CI: 2.09–5.17) (Table 5).

**Table 5:** Prevalence and factors associated with hospitalization in victims of RTA injuries. National Health Survey, Brazil, 2013.

	Prevalence of hospitalization (n = 142)			Bivariate analysis			Multivariable analysis^3^		
Variables	n/Total	%	(95% CI)^1^	Prevalence Ratio	(95% CI)^1^	p-value^2^	Adjusted Prevalence Ratio	(95% CI)^1^	p-value^2^
**Sex**									
Female	45/572	7.9	(4.6–13.3)	1.00			1.00		
Male	97/1,268	7.6	(5.6–10.3)	0.96	(0.52–1.78)	0.914	0.98	(0.53–1.80)	0.963
**Age (years)**									
18–29	41/798	5.2	(2.9–9.1)	1.00					
30–39	49/527	9.3	(5.8–14.4)	1.78	(0.85–3.71)	0.121	2.13	(0.92–4.94)	0.076
40–59	44/409	10.8	(7.1–16.1)	2.07	(1.02–4.20)	0.041	2.46	(1.16–5.23)	0.019
≥ 60	8/106	7.1	(2.8–16.6)	1.36	(0.47–3.95)	0.562	1.90	(0.72–5.3)	0.193
**Race/skin color (self-report)**									
White	63/758	8.3	(5.1–13.2)	1.00			1.00		
Brown	17/198	7.1	(5.2–9.7)	0.85	(0.48–1.52)	0.604	0.72	(0.38–1.37)	0.329
Black	61/862	8.3	(4.0–16.4)	1.00	(0.42–2.35)	0.995	1.03	(0.43–2.45)	0.936
Other (Asian and Brazilian Native)	1/22	5.1	(0.8–26.6)	0.61	(0.09–4.00)	0.612	0.63	(0.09–4.69)	0.219
**Schooling**									
Higher education or post-graduation	18/212	8.4	(4.5–15.3)	1.00			1.00		
High school	45/715	6.3	(3.2–12.0)	0.75	(0.30–1.86)	0.542	0.83	(0.37–1.87)	0.663
Elementary School	37/345	10.5	(6.3–16.9)	1.24	(0.58–2.64)	0.571	1.22	(0.59–2.51)	0.578
Less than elementary school	42/568	7.5	(5.1–10.9)	0.89	(0.43–1.82)	0.754	0.65	(0.33–1.28)	0.219
**Married/partner**									
Yes	83/999	8.3	(6.1–11.1)	1.00					
No	59/841	7.0	(4.3–11.3)	0.85	(0.48–1.50)	0.586			
**Macro-region**									
Southeast	42/633	6.6	(3.7–11.4)	1.00			1.00		
South	18/255	7.2	(3.7–13.5)	1.08	(0.45–1.66)	0.851	1.02	(0.41–2.54)	0.964
Midwest	21/193	11.0	(7.4–16.1)	1.66	(0.84–3.30)	0.143	1.53	(0.77–3.01)	0.217
Northeast	49/542	8.9	(5.2–14.7)	1.34	(0.62–2.88)	0.450	1.41	(0.59–3.35)	0.434
North	12/217	5.7	(3.7–8.7)	0.86	(0.42–1.75)	0.684	0.96	(0.46–2.00)	0.923
**Current drinker**									
No	90/1,053	8.5	(6.0–12.0)	1.00					
Ye	52/787	6.6	(4.4–10.0)	0.78	(0.45–1.33)	0.369			
**Binge drinking**									
No	100/1,340	7.4	(5.3–10.2)	1.00					
Yes	42/500	8.5	(5.3–3.3)	1.14	(0.64–2.0)	0.649			
**Drinking and driving**									
No	128/1,581	8.1	(6.0–10.7)	1.00					
Yes	14/259	5.4	(3.0–9.7)	0.67	(0.34–1.29)	0.236			
**Area of residence**									
Capital	41/408	10.0	(7.1–13.8)	1.00					
Metropolitan region	18/213	8.5	(3.7–18.6)	0.85	(0.35–2.06)	0.726			
Others	83/1,219	6.8	(4.6–9.9)	0.68	(0.41–1.13)	0.141			
**Type of area**									
Urban	123/1,568	8.0	(5.8–10.6)	1.00					
Rural	18/272	6.7	(4.1–10.9)	0.85	(0.47–1.51)	0.583			
**Modes of transport**									
Car occupant	17/496	3.5	(1.9–6.4)	1.00			1.00		
Motorcycle occupant	100/1,069	9.3	(6.6–13.0)	2.64	(1.32–5.38)	0.006	2.91	(1.43–5.93)	0.003
Pedestrian	14/89	15.2	(8.4–26.1)	4.31	(1.88–9.90)	0.001	3.63	(1.78–7.38)	< 0.001
Others	11/186	5.9	(2.6–12.9)	1.66	(0.60–4.57)	0.320	1.78	(0.66–4.80)	0.250
**Prehospital care**									
No	93/1601	5.8	(4.0–8.3)	1.00					
Yes	49/239	20.5	(13.8–29.3)	3.52	(2.09–5.95)	<0.001	3.29	(2.09–5.17)	< 0.001

^1^95% Confidence Interval

^2^Chi-square Wald’s test

^3^Model adjusted by sex, age, race/skin color, schooling, macro-region, modes of transport, and Prehospital care.

For the outcome sequelae and/or disability resulting from RTA injuries, the following associated factors were identified: age between 30 and 39 years (aPR: 1.83; 95% CI: 1.09–3.05) or 40 and 59 years (aPR: 2.06, 95% CI: 1.28–3.32); being a motorcycle occupant (aPR: 3.17, 95% CI: 1.73–5.80), pedestrian (aPR: 3.20, 95% CI: 1.50–6.81) or other mode of transport (aPR: 2.83; 95% CI: 1.31–6.09) and having received PHC (aPR: 2.95 95% CI: 1.97–4.41) (Table 6).

**Table 6:** Prevalence and factors associated with the presence of sequelae and/or disability in victims of RTA injuries. National Health Survey, Brazil, 2013.

Variables	Prevalence of sequelae and/or disability (n = 278)			Bivariate analysis			Multivariable analysis^3^		
	n/Total	%	(95% CI)^1^	Prevalence Ratio	(95% CI)^1^	p-value^2^	Adjusted Prevalence Ratio	(95% CI)^1^	p-value^2^
**Sex**									
Female	106/572	18.6	(13.5–25.0)	1.00					
Male	172/1.268	13.6	(10.7–17.0)	0.72	(0.49–1.07)	0.107	0.72	(0.49–1.05)	0.095
**Age (years)**									
18–29	78/798	9.8	(6.6–14.2)	1.00					
30–39	97/527	18.4	(13.4–24.6)	1.87	(1.15–3.05)	0.012	1.83	(1.09–3.05)	0.020
40–59	87/409	21.3	(15.4–28.7)	2.17	(1.32–3.56)	0.002	2.06	(1.28–3.32)	0.003
≥ 60	16/106	15.3	(8.2–26.7)	1.55	(0.76–3.16)	0.220	1.72	(0.91–3.23)	0.094
**Race/skin color (self-report)**									
White	103/758	13.5	(9.6–18.8)	1.00					
Brown	24/198	12.3	(6.5–22.2)	1.28	(0.84–1.93)	0.238	1.07	(0.69–1.67)	0.637
Black	149/862	17.3	(13.6–21.8)	0.91	(0.45–1.84)	0.796	0.84	(0.43–1.67)	0.730
Other (Asian and Brazilian Native)	2/22	8.8	(1.4–38.8)	0.65	(0.11–3.74)	0.631	0.55	(0.10–2.76)	0.468
**Schooling**									
Higher education or post-graduation	32/212	15.0	(7.4–28.1)	1.00					
High school	95/715	13.3	(9.4–18.6)	0.88	(0.41–1.92)	0.764	0.98	(0.52–1.82)	0.950
Elementary School	42/345	2.1	(8.0–17.8)	0.80	(0.37–1.75)	0.583	0.73	(0.37–1.43)	0.365
Less than elementary school	109/568	19.3	(14.8–24.7)	1.28	(0.61–2.67)	0.506	0.93	(0.51–1.70)	0.819
**Married/partner**									
Yes	178/999	17.9	(14.3–22.1)	1.00			1.00		
No	100/841	11.9	(8.4–16.5)	0.66	(0.44–0.99)	0.047	0.78	(0.54–1.13)	0.199
**Macro-region**									
Southeast	79/633	12.5	(8.0–19.0)	1.00					
South	31/255	12.1	(7.3–19.3)	0.96	(0.50–1.85)	0.919	0.95	(0.53–1.70)	0.878
Midwest	31/193	16.2	(11.3–22.5)	1.29	(0.74–2.24)	0.362	1.08	(0.66–1.76)	0.753
Northeast	102/542	18.8	(13.8–25.1)	1.50	(1.88–2.54)	0.128	1.29	(0.81–2.08)	0.276
North	35/217	16.2	(11.3–22.7)	1.29	(0.74–2.26)	0.362	1.19	(0.71–2.01)	0.494
**Current drinker**									
No	178/1.053	17.0	(13.2–21.5)	1.00					
Yes	100/787	12.7	(9.5–16.7)	0.74	(0.51–1.08)	0.122			
**Binge drinking**									
No	216/1.340	16.1	(12.9–19.9)	1.00					
Yes	62/500	12.5	(8.6–17.7)	0.77	(0.51–1.18)	0.237			
**Drinking and driving**									
No	249/1.581	15.8	(12.9–19.2)	1.00			1.00		
Yes	29/259	11.2	(6.9–17.6)	0.71	(0.43–1.17)	0.178	0.84	(0.53–1.32)	0.462
**Macro-region**									
Southeast	79/633	12.5	(8.0–19.0)	1.00					
South	31/255	12.1	(7.3–19.3)	0.96	(0.50–1.85)	0.919	0.95	(0.53–1.70)	0.878
Midwest	31/193	16.2	(11.3–22.5)	1.29	(0.74–2.24)	0.362	1.08	(0.66–1.76)	0.753
Northeast	102/542	18.8	(13.8–25.1)	1.50	(1.88–2.54)	0.128	1.29	(0.81–2.08)	0.276
North	35/217	16.2	(11.3–22.7)	1.29	(0.74–2.26)	0.362	1.19	(0.71–2.01)	0.494
**Area of residence**									
Capital	53/408	13.1	(10.2–16.6)	1.00					
Metropolitan region	30/213	14.0	(8.0–23.3)	1.06	(0.59–1.91)	0.830			
Others	195/1.219	16.0	(12.5–20.3)	1.22	(0.87–1.72)	0.242			
**Type of area**									
Urban	233/1.568	14.9	(12.0–18.3)	1.00					
Rural	45/272	16.5	(11.1–23.8)	1.10	(0.71–1.71)	0.651			
**Modes of transport**									
Car occupant	32/496	6.5	(3.1–13.1)	1.00			1.00		
Motorcycle occupant	192/1.069	18.0	(14.3–22.2)	2.78	(1.29–5.96)	0.008	3.17	(1.73–5.80)	< 0.001
Pedestrian	23/89	26.2	(16.8–38.5)	4.07	(1.75–9.43)	0.001	3.20	(1.50–6.81)	0.003
Others	31/186	16.8	(10.0–26.8)	2.60	(1.07–6.29)	0.034	2.83	(1.31–6.09)	0.008
**Prehospital care**									
No	191/1601	12.0	(9.6–14.8)	1.00					
Yes	87/239	36.3	(25.7–48.5)	3.03	(2.06–4.47)	<0.001	2.95	(1.97–4.41)	< 0.001

^1^95% Confidence Interval

^2^Chi-square Wald’s test

^3^Model adjusted by sex, age, race/skin color, schooling, married/partner, macro-region, drinking and driving, modes of transport, and prehospital care.

## Discussion

The study showed that less than one third of RTA victims (13.0%) received prehospital care at the site of the accident. The percentage of victims hospitalized for a minimum of 24 hours was 7.7%. The prevalence of sequelae and/or disability was 15.1%.

A study conducted in Yemen, Arabia, showed that 71% of RTA victims had been taken to hospitals by a taxi and only 13% of the victims had been transported by ambulances [17]. A study in New Delhi, India, with emergency hospital data showed that ambulances were used to transport the victims in only 14.6% of cases of RTA injuries [18]. In West African Malawi, access to prehospital care for RTA victims is almost non-existent, and rapid transport to the hospital is generally prioritized, regardless of whether or not prehospital care is provided. This is due to limited knowledge of first aid measures and lack of access to basic equipment for provision of care [19].

In Brazil, PHC was provided more frequently by the SAMU, which can be explained by the rapid expansion of this service in Brazil, making it the main reference for emergency prehospital care in the country’s capitals [20]. However, the time elapsed between the accident and the first care measure for most of the victims was greater than 60 minutes. A study conducted in five Brazilian capitals [20] and in São Paulo showed a shorter time than that found in the present study [7]. International studies, such as those from the United States of America (USA) [21] also showed lower times, while Pakistan [22] and Mexico [23] presented a similar time to the one found in the present study.

The recommended time for first care, described in international studies, is 60 minutes for starting emergency care. This time is called the Golden Hour and has a major influence on the chances of survival of the injured victims [9, 24, 25].

A three-year review of RTA victims treated at a hospital in Nigeria showed that only 24.0% of the victims arrived to the emergency room within one hour after the accident, while one-third arrived between one and six hours; 55.4% were taken by relatives and 21.4% by volunteers, and only 2.3% of RTA victims received PHC at the site of the accident [26]. These results show the lack of rapid response of the health system, leading to a high percentage of death before arriving at the hospital. Prehospital mobile care within the first hour and provided by trained professionals with appropriate equipment can prevent deaths and serious sequelae [23]. In developed countries, there is usually an emergency number that can be dialed by any person, while in the middle- and low-income countries, this modality of care is not widespread, leading to iniquities between countries as to the severity of the injuries [1, 27].

A factor that may explain the time for PHC to reach the site of the accident above the ideal 60 minutes may be the current model of urban expansion of cities that stimulate urban densities in areas far from the central nucleus, where, in general, the health trauma and prehospital care centers are located. A study conducted in the USA showed that the likelihood of delay of ambulances is almost twice as high in municipalities with urban expansion whose characteristics include low construction density, poor street connectivity, and separation of residential and industrial development compared to municipalities that exhibit characteristics of intelligent growth [28]. This has an impact on the choice of some victims to use the private transport to displace the individual to the urgency and emergency units. One way to reduce the time of displacement of the mobile urgency care service is the decentralization of hospital services and of mobile health service bases in the regions of the cities [29, 30].

Studies that evaluate the coverage and quality of prehospital mobile services used to assist RTA victims are scarce. A contributing factor is the low coverage of these services and the lack or poor quality of records of information related to the victims, the characteristics of the injuries, and the health care procedures adopted [31]. The standardization and collection of this information in prehospital mobile services is fundamental to guide prevention actions and for reduction of injuries caused by RTA [6].

Motorcycle occupants were the group of victims who most received PHC in Brazil. Similarly, to the present study, a study that analyzed occurrence reports of PHC in the state of Espírito Santo, Southeastern Brazil, showed that most users of PHC service were motorcyclists [32]. Other study carried out in São Paulo showed that the highest percentage of RTA victims who used prehospital mobile services were pedestrians and motorcycle drivers or passengers [7]. A study carried out in Mexico also showed similar results; motorcyclists followed by occupants of motor vehicles and pedestrians were the users of PHC service as a result of RTA injuries [23]. This greater use by motorcyclists and pedestrians is due to the greater vulnerability of these two traffic users, who also present a greater severity of injuries in cases of RTA [33].

In the present study, although pedestrians received a higher percentage of PHC, no statistically significant association was found as in motorcycle occupants, probably because of the small number of pedestrians in the sample of the 2013 NHS, or because pedestrians, rather than waiting for on-site service, are transported quickly by vehicles of people who hit them or who passed in the road at the time of the RTA, or yet they are left in the place after the driver that caused the accident escapes [15, 23].

Victims living in the North, Northeast and rural areas had lower rates of PHC, showing low coverage in those regions and in rural areas. These inequalities reflect the low coverage or lack of decentralization of PHC centers to rural areas [29, 34].

With respect to the lower use of PHC in rural areas when compared to urban areas, the literature is scarce. Similar results were found in studies on mortality due to RTA, such as one conducted in China that showed that a high percentage of deaths due to traffic injuries occurred in rural areas, while 24% occurred in urban areas [35]. This may be related to lack of timely on-site care. Another study carried out in South Africa, Cape Town, Western Cape, showed that more than half of the deaths from RTA occurred in rural areas without PHC in the first hour of the accident [25]. Although the estimated coverage of SAMU is 53.9% of the Brazilian population [30], it was observed in this study that the rural population was not covered by this service. This low coverage can be attributed to the dispersion of rural areas and the difficult operationalization of PHC services. The realization of population studies that portray the dimension of this reality and that identify possible clusters of accidents in rural areas may guide the size and the priority areas for the expansion of this model of care in Brazil.

Other studies point to other limitations that affect the delay and quality of PHC for victims and are related to the absence of predefined emergency medical services, the provision of poor first aid measures, professionals with insufficient skills, lack of training, insufficient material and human resources, and traffic jam [36].

As for hospitalization of RTA victims, a percentage of 7.7% was observed in Brazil in the year 2013. Results of the Survey of the Surveillance System for Violence and Accidents (VIVA) performed in emergency and urgency services of 23 capitals and the Federal District in 2014 showed that 22.3% of the victims were referred for hospitalization [37]. International studies as the one performed in Mexico [23] from 2012 to 2014 showed that 27.1% of RTA victims were hospitalized for 24 hours or more. In Catalonia [Spain] [38], in 2013, 12.9% of the patients with RTA injuries received care in the emergency department and 10.2% were hospitalized. In a study conducted in Buenos Aires, 18% of the victims required hospitalization [39]. The hospitalization of RTA victims portrays the severity of the event and is linked to a considerable part of the costs resulting from this condition [5, 33].

Regarding the characteristics of the hospitalized victims, a high percentage was observed to be in the age range from 40 to 59 years. Results of a study carried out in three reference hospitals that provide care for trauma in the city of São Paulo showed that the mean age was 26.2 years for hospitalized RTA victims [40] and a study carried out in Iran revealed that men between the ages of 15 and 29 years, and 30 to 44 years were among the mostly hospitalized groups [41]. Data from the HIS/SUS in 2013 showed that the individuals aged 20–39 years were the main RTA victims [33]. This difference is related to the fact that the present study interviewed individuals in the residences rather than individuals who entered trauma care services; motorcycle users, who have more severe injuries, are generally younger. In the household approach, the spectrum of severity may include milder injuries, or injuries that led to death before care in emergency units [23].

It was observed that being a motorcycle occupant and pedestrian was associated with a higher prevalence of hospitalization. Study with data from 2013 in Brazil showed that more than half of the hospitalizations for RTA were motorcyclists [51.9%], followed by pedestrians [25.8%]. Moreover, motorcyclists were the victims with the longest hospital stay [33]. In Buenos Aires, most of the total number of hospitalization due to RTA resulted from injury caused by motorcycle accidents [39]. A study carried out in Thailand, a country with a high rate of motorcycle use, showed that among the hospitalizations due to RTA in Bangkok over a one-year period, the number of those caused by motorcycle accidents was greater than that caused by car accidents [42]. In India, 13.3% of the motorcycle occupants who were injured in an RTA were hospitalized [43]. A study carried out in Iran in 2011 showed that motorcyclists were responsible for 39.2% of hospitalizations [41].

In the present study, it was observed that victims who received prehospital care had higher prevalence of hospitalization and sequelae and/or disability. A similar result was found in a study carried out in two cities in Mexico, Guadalajara and Leon, which found that victims who received prehospital care were more likely to have prolonged hospitalization, disability or death [23]. This may be related to the fact that RTA that result in more serious injuries are prioritized for on-site care, and are also more likely to lead to hospitalization, disability, and sequelae [22, 23, 27]. Deficiencies resulting from traffic injuries represent a significant burden on low- and middle-income countries. They threaten economic productivity [44].

The prevalence of sequelae and/or disabilities in RTA victims in the present study was 15.1%. A study with data from 2000 to 2013 showed that 23.5% of RTA victims had a diagnosis suggestive of physical sequelae in Brazil [45]. Data from the 2014 VIVA Survey showed that the presence of some type of sequelae and/or disability (either physical, mental, visual, auditory, and other deficiencies/syndromes) was reported in 2.2% of RTA victims [37]. A study conducted in Mexico between 2012 and 2014 found that 5.94% of RTA victims had a diagnosis of permanent disability [23]. A study conducted in China estimated a prevalence of 1.12 individuals per 1,000 inhabitants with sequelae resulting from RTA [46]. Another study in Yemen found a percentage of sequelae and/or disability close to 40% [17]. In the National Survey on Disability in Spain in 2008, 2.1% of the disabilities were the result of RTA [47].

The ages between 30 and 39 years and 40 and 59 years were considered factors associated with the presence of sequelae and/or disability, like the study carried out in the USA, which observed a higher prevalence of disability related to traffic accidents in the age group of 35 to 64 [48]. In the Spanish survey, people between 31 and 64 years old had higher frequencies of RTA-related disability [47, 49]. This association can be explained by the physical vulnerability of older people that predisposes them to higher risk of serious and fatal injuries and greater chance of sequelae [23, 36, 48].

As for the modes of transport, the occupants of motorcycles, other modes of transport, and pedestrians had the highest prevalence of sequelae and/or disability. In the 2014 VIVA survey, motorcyclists were the ones who presented the highest percentage of hospitalization for RTA injuries with a diagnosis of sequelae [37]. A study carried out in four low-income countries (Sierra Leone, Rwanda, Nepal, and Uganda) showed that 38.5% of RTA victims had disabilities, more frequently pedestrians and motorcyclists [44]. A study in Belgium showed that the prevalence of disability was higher among motorcyclists and cyclists [50]. The higher prevalence of disability in these groups of victims is explained by the high proportion of head injury in cyclists and lower limb injuries in motorcyclists [51].

The only association found in the three outcomes among the occupants of other modes of transport was with the presence of sequelae and/or disability. A hypothesis for this association would be that cyclists were included in this category, and just like motorcyclists and pedestrians, they are vulnerable road users and may be affected by more serious accidents [6, 44].

The fact that higher prevalence rates of the three outcomes studied were found among motorcycle occupants can be explained by the rapid increase in the production and commercialization of this mode of transport in Brazil from the 2000s onwards. This exponential growth of the motorcycle fleet in Brazil was prompted by the incentive given to motorcycle manufacturers, as well as credit facilitation for acquisition of motorcycles, and the precariousness of collective public transportation in the country. These aspects have caused a significant number of people to adopt motorcycles as a mode of transport, to travel and to work [4, 5, 33]. Consequently, there has been an increase in the number of RTA involving this type of vehicle and a dizzying increase in mortality rates linked to motorcycle accidents in Brazil in the last decade [34]. Since most road users in low- and middle-income countries are pedestrians, cyclists and motorcyclists, the implementation of a traffic safety policy prioritizing these users is paramount [1, 6, 44].

Among the limitations of this study we must mention the probable presence of memory bias of the interviewed adults, which may influence the quality of the data collected during the interviews. Another issue is survival bias, which is a common limitation of prevalence studies. The absence of a greater number of independent variables that could be analyzed in the study such as car or motorcycle ownership, occupation, location, day of the week and time when the traffic accident happened, may have reduced the ability to identify the factors associated with the outcomes studied. Another limitation is the non-inclusion of RTA victims who did not present lesions or injuries of low severity; this may overestimate the prevalence of the outcomes. Another limitation of the cross-sectional study is that it does not allow the establishment of cause and effect relationships between predictor and outcome variables. And finally, the limitation of not having specific information about the type of sequelae and/or disability resulting from the traffic accident, which made it difficult to compare our findings with other studies.

## Conclusion

In conclusion, data from the NHS allowed to evaluate the factors associated with PHC, hospitalization and presence of sequelae and/or disability in the victims of RTA. Our study estimated the prevalence of RTA injuries that include the serious and minor injuries collected in a population sample and not from people who demanded emergency care services as most published studies have done. The study filled this knowledge gap and enabled an estimate of the magnitude and factors associated with PHC, hospitalization and presence of sequelae and/or disability in RTA victims in the Brazilian population.

From the findings of this study, it is worth mentioning that surveillance of accidents and violence, the adoption of educational and legislative measures for road safety that contribute to the reduction of morbidity and mortality due to this condition, is fundamental. It is also important to invest in mobile and PHC quality, especially in the Northeast and North regions and in the rural area of the country.

This study may guide the implementation of interventions that prioritize the population exposed to the highest risk of RTA injuries and with less access to prehospital and hospital care services in Brazil. Initiatives to prevent injuries that lead to hospitalization and sequelae and/or disability are urgent due to the heavy burden of morbidity and mortality resulting from RTA in the country. Furthermore, the study may guide the planning of prehospital and hospital care services in the Brazilian regions, enabling access to timely prehospital care, and may help to alleviate the burden of RTA. Inter-sectoral structural measures led by the federal government, together with states and municipalities, can help reduce the magnitude of the incidence of RTA in Brazil and address regional inequalities in the distribution of RTA. In addition, interventions aimed at road safety for vulnerable road users, such as pedestrians and motorcyclists, and the population groups most likely to experience RTA, as identified in this study, are vital to reduce inequalities within the population.

It is worth emphasizing the importance of traffic safety regulations with respect to surveillance, penalties, strengthening traffic safety management institutions, and PHC for RTA victims. These measures can reduce the chances of occurrence of RTA and can also reduce the severity of the injuries and the number of deaths, especially among the most vulnerable traffic users.

Finally, new research directions are suggested: population-based surveys to investigate the magnitude of PHC, hospital care and presence of sequelae and/or disability in the victims of RTA; investigation of variables associated with these outcomes, in addition to sociodemographic and behavioral ones, such as quality of care, health care network, among others; sensitivity analysis to analyze the associated factors according to region, age, sex and education.
